# CARD9 in host immunity to fungal, bacterial, viral, and parasitic infections: An update

**DOI:** 10.3389/fmicb.2022.1021837

**Published:** 2022-11-09

**Authors:** Ang Hu, Zeming Hu, Haohong Zou, Jiankang Zhang, Dongliang Zhang, Hao Wang, Jianing Zhong, Bin Chen

**Affiliations:** ^1^Department of General Surgery, The First Affiliated Hospital of Gannan Medical University, Ganzhou, China; ^2^Key Laboratory of Prevention and Treatment of Cardiovascular and Cerebrovascular Diseases, Ministry of Education, Gannan Medical University, Ganzhou, China; ^3^School of Medicine, Ningbo University, Ningbo, China

**Keywords:** CARD9, fungi, bacteria, viruses, parasites

## Abstract

Microbial infection, caused by fungi, bacteria, viruses, and parasites, significantly contributes to the global death burden and health costs. The innate and adaptive immune systems orchestrate a multifaceted signaling response to invading pathogens as the human antimicrobial system. In this process, caspase recruitment domain-containing protein 9 (CARD9) emerges as a critical intermediary adaptor molecule to participate in regulating a series of antimicrobial immune reactions. Previous publications have confirmed that CARD9 plays a crucial role in fungal, bacterial, viral, and parasitic infections. In this study, we aim to provide an update on the recent clinical and basic studies where the mechanism and function of CARD9 have been further studied and understood. In addition, we summarize the latest treatment and prevention strategies based on CARD9 and discuss the current perspectives and future direction of CARD9.

## Introduction

**Figure 1:**
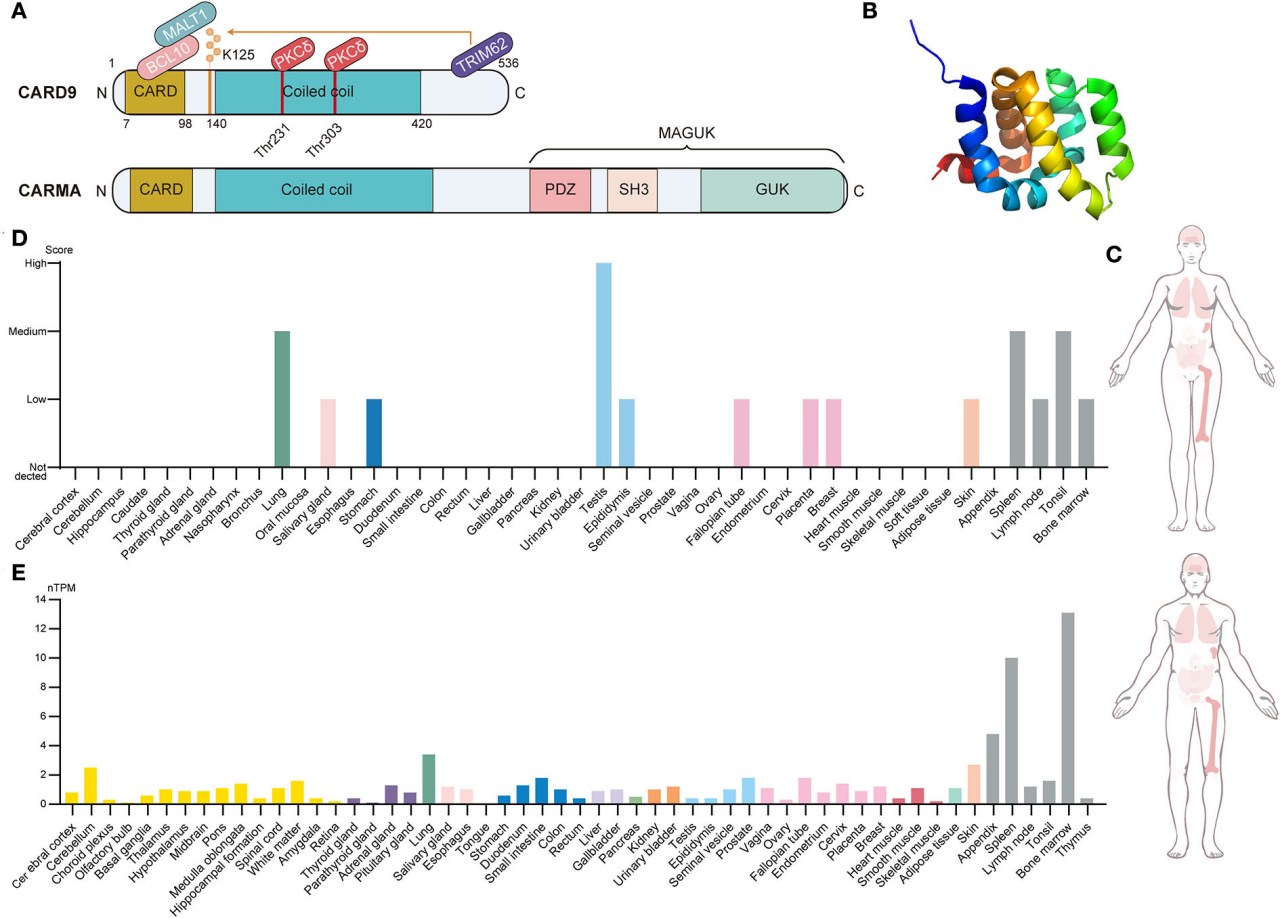
Brief introduction to CARD9. **(A)** Structural features, protein-interacting domains, and post-translational modification sites of CARD9 and CARMA. CARD9 and CARMA family have a similar structure in which the N-terminal CARD domain, the coiled-coil domain, and the C-terminal tail are shared. Apart from these, CARMA still contains the C-terminal MAGUK region, which includes the PDZ domain, SH3 domain, and GUK domain. PKCδ-mediated phosphorylation (Thr 231 and Thr 303) and TRIM62-mediated ubiquitination (Lys 125) are required for the activation of CARD9. BCL10 interacts with activated CARD9 on the homologous CARD domain, and then they bind to MALT1 to induce the assembly of the trimolecular complex. **(B)** The NMR solution structure of the CARD9 CARD. Image from the RCSB PDB (RCSB PDB accession code PDB 6E26). **(C)** Integrated RNA and protein expression of CARD9 in the various normal tissues of a different gender. The color shade of the tissues represents the extent of expression (Data from The Human Protein Atlas). **(D)** CARD9 protein expression overview categorized by the system. Color coding is based on tissue groups that contain tissues with functional features in common (Data from The Human Protein Atlas). **(E)** CARD9 RNA expression overview categorized by the system. Color coding is based on tissue groups that contain tissues with functional features in common. nTPM represents the normalized expression of RNA (Data from The Human Protein Atlas). MAGUK, membrane-associated guanylate kinase; SH3, Src Homology 3; GUK, Guanylate Kinase; MALT, mucosa-associated lymphoid tissue lymphoma; BCL10, B cell lymphoma 10; PKCδ, protein kinase Cδ.

Caspase recruitment domain-containing protein 9 (CARD9), a novel member of the CARD family, was originally discovered through Millennium Pharmaceuticals' proprietary database searches with previously known CARD family proteins (Bertin et al., 2000). The CARD family is the second largest family of the death domain (DD) superfamily and is characterized by the presence of a caspase-associated recruitment domain (Kao et al., 2015). Of these, the protein composition of CARD9, including the N-terminal CARD domain, coiled-coil domain, and C-terminal tail, is shown in Figure 1 (Ruland and Hartjes, 2019). The N-terminal CARD region (amino acids 7–98) is a protein–protein interaction module that binds to CARD-containing apoptosis proteins to mediate intracellular signaling cascades and regulate cell apoptosis responses to an altered extracellular environment (Bertin et al., 2000). The coiled-coil region (amino acids 140–420) takes charge of the process of protein oligomerization, forming heptad repeats labeled “*abcdefg*,” with *a* and *d* denoting the hydrophobic residues (Hara and Saito, 2009). The C-terminal tail is the designated site where ubiquitin ligase TRIM62 interacts with CARD9. TRIM62 is responsible for the ubiquitination of lysine 125, which is a requisite for the activation of CARD9 (Cao et al., 2015). CARD-containing membrane-associated guanylate kinase (MAGUK/CRAMA) proteins family, including CARD11 (CARMA1), CARD10 (CARMA2), and CARD14 (CARMA3), has been proposed to have a similar function to CARD9 (Bertin et al., 2001; Wang et al., 2001). However, the difference lies in protein structure because CARD9 lacks the C-terminal MAGUK region, which contains PDZ, Src Homology 3, and the Guanylate Kinase domain (Jiang and Lin, 2012). CARD9 is a protein of 536 amino acids with a predicted molecular weight of 62.3 kDa and has been mapped to the 9q34.3 chromosomal region (Bertin et al., 2000). Figure 1 provides the CARD9 protein and mRNA expression of various human tissues, indicating that high expression is mainly observed in immune-related tissues. At the cellular level, CARD9 is abundantly expressed in myeloid cells, such as monocytes, neutrophils, macrophages, and dendritic cells (Hsu et al., 2007).

Generally, CARD9 acts as a crucial downstream member of pattern recognition receptors (PRRs) and mediates a series of inflammatory cascades against invasive fungi, bacteria, viruses, and parasites. Mutation of the CARD9 gene correlating with lower expression and function loss is an autosomal recessive primary immunodeficiency disorder, and it predisposes individuals to microbial infection. The downstream of PRRs, PRRs/Syk/CARD9, is one of the most classic signaling pathways. When PRRs recognize specific microbial pathogens, PPRs recruit spleen tyrosine kinase (Syk) to activate the protein kinase Cδ (PKCδ), which can coordinate with VAV to phosphorate CARD9 at positions Thr 231 and 303 in the coiled-coil protein domain. Phosphorated CARD9 induces the formation of multiple protein complexes termed CBM in the cytoplasm, which consists of CARD9, B cell lymphoma 10 (BCL10), and mucosa-associated lymphoid tissue lymphoma (MALT1) and acts as a signaling integration center (Hartjes and Ruland, 2019). Under the control of CBM, the nuclear factor-κB (NF-κB) and mitogen-activated protein kinase (MAPK) signaling pathways are initiated to regulate multiple cellular processes (described below) in downstream cascades (Drummond et al., 2018). A more detailed introduction to the CARD9 signaling network can be seen in the studies by Roth and Ruland (2013) and Wang et al. (2020). At present, the relationship between CARD and host immunity has been extensively studied. Many emerging studies have broadened the scope of the known mechanisms and improved our understanding of the function of CARD9 in microbial infection. Herein, we briefly describe previous findings and review recent studies to further elucidate the inner relationship between CARD9 and infection immunity.

## Fungi

It is well acknowledged that CARD9 and its related signaling network play a pivotal role in the immunology of fungal infection (Drummond et al., 2018). Mechanistically, the C-type lectin receptor (CLR) family, including Dectin-1, Dectin-2, Dectin-3, and Mincle, initiates the signaling cascades against the fungal invasion through ligand-receptor binding. Studies have mainly concentrated on Dectin-1, which can be recognized by fungus components, although the downstream Syk-dependent pathway identifies all these receptors. Indeed, the Syk/CARD9/CBM cascade and the following NF-κB and MAPK signaling pathways are defined as the main effector signaling networks against fungal invasion.

Earlier studies have found that a deficiency of CARD9 makes many fungi susceptible (Wu et al., 2018), such as *Cryptococcus neoformans* (Yamamoto et al., 2014) and *Phialophora verrucose* (Wu et al., 2016). However, recent studies have further broadened this range by proving the susceptibility to *Aspergillus* (Zhang et al., 2021), *Pneumocystis* (Kottom et al., 2020b), *Mucor irregularis* (Sun et al., 2021), and *Rhizopus arrhizus* (Sun et al., 2019). For instance, Guo et al. (2019) reported that individuals with homozygous CARD9 alleles (C/C) were more susceptible to *Pallidocercospora crystalline* compared to those with heterozygous (G/C) or wild-type (G/G) CARD9 alleles. Another report identified that 17 patients with deep dermatophytosis from Tunisian, Algerian, and Moroccan families displayed autosomal recessive CARD9 deficiency (Lanternier et al., 2013). Furthermore, Rieber et al. (2016) showed that CARD9 deficiency was the first known inherited or acquired a status that was predisposed to extrapulmonary aspergillosis with sparing of the lungs.

Given the association between CARD9 and fungal susceptibility, more and more biologists and medical scientists are struggling to elucidate this intricate mechanism. Recently, Kottom et al. (2020a) performed a comprehensive analysis of the susceptibility of CARD9 KO and immunocompetent hosts to *Pneumocystis*. They demonstrated that this defect was attributable to a compromised proinflammatory response, in which CARD9^−/−^macrophages could not perform cell differentiation and express polarization markers, thereby failing to upregulate the expression of CLRs. Intriguingly, the CARD9 KO mice not only exhibited an intact T-helper (Th) cell cytokine but displayed a normal survival rate, the same as that of wild-type (WT) mice. The probable explanation is that the modulation of Th cytokine may be CARD9-independent, and low-level inflammatory response may ameliorate lung injury. Wang et al. (2018) identified a novel CARD9 mutation (p.S23X) in patients susceptible to *dematiaceous fungi*. They found the impairment of proinflammatory cytokine and chemokine production, neutrophil recruitment, pathway activation (NF-κB and MAPK), and Th-associated (Th 17 and Th 22) responses in patients with CARD9 deficiency. Remarkably, neutrophil phagocytosis and reactive oxygen species (ROS) production remained normal. Similarly, in a study of susceptibility to *M. irregularis*, the release of neutrophil extracellular traps (NETs) and the expression of IL-6 and TNF-α showed an impaired state in neutrophils, whereas intact phagocytosis and ROS production were observed (Sun et al., 2021). Different from the impaired neutrophil antifungal immunity, the functions of phagocytosis and ROS production are seemingly conflicting. Indeed, these results were in line with the previous studies in which the same situation of *Phialophora verrucosa* and *Candida albicans* infection were reported. This may be because both of these functions are free of the control of CARD9 (Gazendam et al., 2014; Liang et al., 2015). Generally, neutrophils constitute the first line of defense against fungal invasion, in which infiltration, phagocytosis, ROS, and NETs participate. Local infection provides the necessary CXC signal to induce neutrophil recruitment indirectly, in which CARD9 has been proven to play a crucial part. Recent studies found that the neutrophil-targeting chemokines CXCL1 and CXCL2 were a low expression in Card9^−/−^ mice, and CARD9 could promote the neutrophils chemotaxis by regulating the production of neutrophil-recruiting IL-1β and CXCL1 in the central nervous system, thereby confirming the importance of CARD9 in neutrophil-induced immune response (Drummond et al., 2019; Sun et al., 2021). In addition to these, the increased susceptibility of Card9^−/−^ mice to *R. arrhizus* and *M. irregularis* were reported as well, and they were attributed to impaired cytokine and chemokine production, NF-κB (p65) activation, and Th cell differentiation (Sun et al., 2019, 2021).

B-cell-mediated humoral immunity acts as another crucial line of antifungal defense, in which CARD9 shows an emerging regulatory role. Recently, Doron et al. (2021) identified *C. albicans* as the antibodies preferentially targeted species and the main trigger of the production of fungicidal IgG. They found that colonization of intestinal fungi depends on CARD9 and CARD^+^CX3CR1^+^ macrophages to regulate the generation of antifungal antibodies, proving that the loss of CARD9 functionality induced by mutation was connected with disturbed IgG reaction. However, *C. albicans* is known as a beneficial commensal in the human gut, although it offers a classic function model of CARD9 in humoral immunity. Further exploration of the humoral immunity of detrimental and invasive fungi is needed to provide more intuitive insights into CARD9 and fungal infection.

Current studies have provided insight into the intricate regulation network and protein interactions of CARD9. For example, Loh et al. (2019) reported that Dok3 coordinated with protein phosphatase 1 (PP1) to dephosphorylate CARD9, ultimately inhibiting the downstream NF-κB and JNK signaling pathway activation and antifungal immune response. Moreover, Tartey et al. (2018) revealed that a previously unknown cross-talk between CARD9 and SHP-1 modulated IL-1α-induced signaling cascade and inflammation response in a mouse model of neutrophilic dermatoses. These studies further enrich the functional mechanisms of CARD9 while indicating its future research direction.

Taken together, CARD9 is an indispensable regulator in antifungal immunity, and CARD deficiency is generally accompanied by fungal susceptibility (new mutations of CARD9 over the past 4 years are listed in Table 1). Potential reasons could be dampened cytokine production, Th-cell differentiation, neutrophil immunity, and pathway activation (Vornholz and Ruland, 2020; Wang et al., 2020; Sheng et al., 2021). Although most of these mechanisms have been documented in previous publications, it is necessary to further explore other fungal species because species-specific mechanisms exist in certain fungi, such as *Candida parapsilosis* and *C. albicans* (Zajta et al., 2021). Notably, an emerging view indicates that Syk has a more eminent impact on anti-Candida immunity than CARD9 in the classic Syk/CARD9 antifungal pathway due to the presence of Syk-dependent but CARD9-independent antifungal signaling (Zajta et al., 2021). Therefore, this viewpoint may prompt a new research direction targeting CARD9 and Syk, which are mutually independent mechanisms.

**Table 1:** New mutations of CARD9 over the past 4 years.

**Mutation**	**Identified year**	**Patient age**	**Patient sex**	**Country**	**Fungus**	**Organ involvement**	**References**
p.V261fs	2018	8	F	Turkey	*Prototheca zopfii*	Digestive tract/mucous membrane	Sari et al., 2018
p.Gln295Ter	2018	17	F	Turkey	*Candida albicans*	Oral/respiratory tract/CNS	Cetinkaya et al., 2018
c.1269+18G>A	2019	31	M	NA	*Trichophyton rubrum*/*Trichophyton violaceum*/*Aspergillus fumigatus*/*Aspergillus flavus*	Skin/Nail/Lymph nodes	Nazarian et al., 2020
Ala586Gly	2019	30	F	China	*Trichophyton rubrum*	Skin/nails	Huang et al., 2019
c.692C>T	2019	46	F	China	*Mucor irregularis*	Skin	Wang et al., 2019
c.905_907delTCT	2019	46	F	China	*Mucor irregularis*	Skin	Wang et al., 2019
c.586A>G (p.K196E)	2021	4	F	Japan	*Exophiala dermatitidis*	Lymph nodes/CNS	Imanaka et al., 2021
c.610C>T (R204C)	2021	Child	M	China	*Talaromyces marneffei*	Skin/lung/liver/lymph nodes/spleen	Ba et al., 2021
Q370X (c.1108C>T)	2021	26	F	India	*Exserohilum rostratum*	Skin	Kalantri et al., 2021
c.A586G	2021	6	M	China	*Alternaria* species	CNS	Lai et al., 2021

F, female; M, male; CNS, central nervous system.

## Bacteria

The antibacterial signaling cascades of CARD9 are initiated by toll-like receptors (TLRs), a type of PRR. TLR2 and TLR4 are two main members of the TLR family that recognizes the components of gram-positive and gram-negative bacteria, respectively. For instance, muramyl-dipeptide (MDP) and peptidoglycan for the former (Akira et al., 2006; Kawai and Akira, 2010), and lipopolysaccharide (LPS) for the latter (Kawai and Akira, 2010). Once recognized, TLRs collaborate with myeloid differentiation primary response 88 (MyD88) to recruit interleukin-1 receptor-associated kinase (IRAK1) and receptor-interacting protein 2 (RIP2) before inducing the assembly of the CBM protein complex (Kobayashi et al., 2002; Dong et al., 2006). Nucleotide-binding oligomerization domain 2 (Nod2) interacts with CARD9 to promote the recognition of MDP and the activation of the JNK and p38 signaling pathways and ultimately regulates the production of inflammatory cytokines against bacterial infection. Meanwhile, classic CLR signaling also participates in antibacterial immunity, which is further proved by several recent studies. Previous publications have revealed that Dectin-2 served as a critical regulator in host immunity against *Streptococcus pneumoniae* infection by affecting phagocytosis of neutrophils but excluding the recruitment of neutrophils (McGreal et al., 2006; Akahori et al., 2016). At present, Ishizuka et al. (2020) found that CARD9 KO mice other than dectin-2 KO mice showed impaired neutrophil recruitment and decreased inflammatory cytokine and chemokine production compared to respective control mice. They indicated that CARD9-mediated signaling was indispensable in anti-*pneumococcal* immunity through modulating neutrophil function and cytokine production, in which both neutrophil phagocytosis and neutrophil accumulation required the participation of CARD9. However, the regulatory signaling of the former was initiated by Dectin-2, while that of the latter was initiated by another non-Dectin-2 CLR or an unidentified CLR. Notably, Mincle may be a promising candidate for this due to its ability to recognize glucosyl-diacylglycerol, a key component of *S. pneumoniae* (Behler-Janbeck et al., 2016). Moreover, Prado Acosta et al. (2021) identified Mincle as a receptor for the surface (S)-layer of *Lactobacillus brevis*. They found that S-layer/Mincle interaction induced a balanced immune response between pro- and anti-inflammatory cytokines through the Mincle/Syk/CARD9 axis. Under this circumstance, the deletion of Mincle, CARD9, and Syk disturbed the balance, resulting in upregulated proinflammatory and downregulated anti-inflammatory cytokines. Meanwhile, an altered CD4^+^ T cell priming capacity was observed under the Mincle knockdown, implying this signaling pathway was also involved in regulating cellular immunity. In most instances, CARD9 acts as a critical downstream adaptor molecule for signal transduction triggered by PRRs. However, PRRs can still initiate CARD9-independent antibacterial signaling, which interweaves with a CARD9-dependent mechanism to exert a bactericidal effect (Akahori et al., 2016). Therefore, further investigation is still necessary to define the different mechanisms of different receptors.

## Viruses

Although the composition of the virus is merely genetic material and essential enzyme, some PRRs, including DC-SIGN, L-SIGN, Langerin, MMR, DCIR, MDL-1, LSECtin, MGL, TLR2, TLR3, TLR4, TLR7, TLR8, and TLR9, has been confirmed to involve in recognition of viral components (Drummond et al., 2018; Zhou et al., 2021). As the classic downstream molecule of PRR signaling, CARD9 has been acknowledged to play a key role in viral infection. For example, CARD9 interacted with DNA sensor Rad50 and dsDNA to induce the assembly of the dsDNA-Rad50-CARD9 complex. It ultimately caused the activation of NF-κB and the generation of a pro-IL-1β response to the virus (Roth et al., 2014). A similar mechanism can also be observed in RNA virus recognition by RIG-I, which, together with MAVS, CARD9, and Bcl-10, for NF-κB activation (Poeck et al., 2010). Recently, Monteiro et al. (2019) recognized Dectin-1, Dectin-2, and Mincle as several potential receptors to interact with the La Crosse virus (LACV). The evidence is that LACV infection of Mincle^−/−^ and CARD9^−/−^ DCs displayed impaired proinflammatory cytokine production, including IL-6 and TNF-α. However, the deletion of CARD9 and Mincle did not alter the elimination of LACV. Therefore, they concluded that the Mincle/CARD9 axis played an indispensable role in early anti-LACV immunity. In parallel, Pavasutthipaisit et al. (2021) reported that CARD9 had a limited effect on early antiviral reactions and encephalomyelitis virus (TMEV) removal. In their study, the knockout of CARD9 eliminated the production of proinflammatory cytokines and the infiltration of T and B cells. It even triggered a temporary increase of TMEV-infected cells in the brain. Moreover, they still confirmed that CARD9 deficiency did not affect the initiation of CD8^+^ T cell response. In a Coxsackievirus B3 (CVB3)-induced viral myocarditis mouse model, CARD9 deletion downregulated the mRNA and protein expression of TGF-β, IL-17A, and BCL-10, thereby ameliorating the CARD9-involved potent inflammation response (Sun et al., 2020). Based on these, a conflicting effect of CARD9 in viral infection is unfolding before our eyes. On the one hand, it can modulate the host's innate immune response to viral infection. On the other hand, it can cause severe viral infection and potential immune-pathological damage.

## Parasites

TLR2, TLR4, TLR9, TLR11, Dinctin-2, SIGNR5, and Mincle have been identified as the prominent sensor of parasite pathogens (Ashour, 2015; Kalantari et al., 2018). SIGNR5 has been reported to coordinate with Dectin-2 and Mincle to initiate the FcRγ-Syk-CARD9 signaling and then promote IL-1β and IL-23 production and Th 17-induced immunity against *Schistosome* Egg (Kalantari et al., 2018). In *Toxoplasma gondii*, Pandori et al. (2019) found that the Syk-CARD9/MALT-1-NF-κB signaling pathway was activated to regulate NLRP3 inflammasome-mediated IL-1β production in human monocytes against pathogens. Intriguingly, IL-1β is released by monocytes in general upon gasdermin-D-induced pyroptotic cell death (Cookson and Brennan, 2001). In contrast, *T. gondii* was shown to initiate the release of IL-1β in a pyroptosis-free and gasdermin-D independently. Moreover, Maknitikul et al. (2018) reported that the CARD9 signaling pathway regulates host immune response induced by hemozoin in *Plasmodium falciparum*-associated acute lung injury, such as the production of IL-1β and the apoptosis of type II alveolar cells. However, a previous publication showed CARD9 did not participate in the *Plasmodium berghei*-induced pathology in experimental cerebral malaria. Their evidence is that similar pathologic features, including impaired blood-brain barrier, elevated proinflammatory response. Brain-infiltrating CD8+ cells were observed in both WT and Card9^−/−^ mice, although the expression of CARD9 increased during *P. berghei* infection (Hafalla et al., 2012). Possible reasons for these ambivalent results lie in the tissue specificity between brain and lung and/or fungal species between *P. falciparum* and *P. berghei*. Studies on parasites are currently in the minority compared to studies on fungi, bacteria, and viruses. As a crucial regulator of host defense, CARD9 is worthy of more attention and investigation within the scope of antiparasitic immunity.

## Emerging infection treatment and prevention based on CARD9

**Figure 2:**
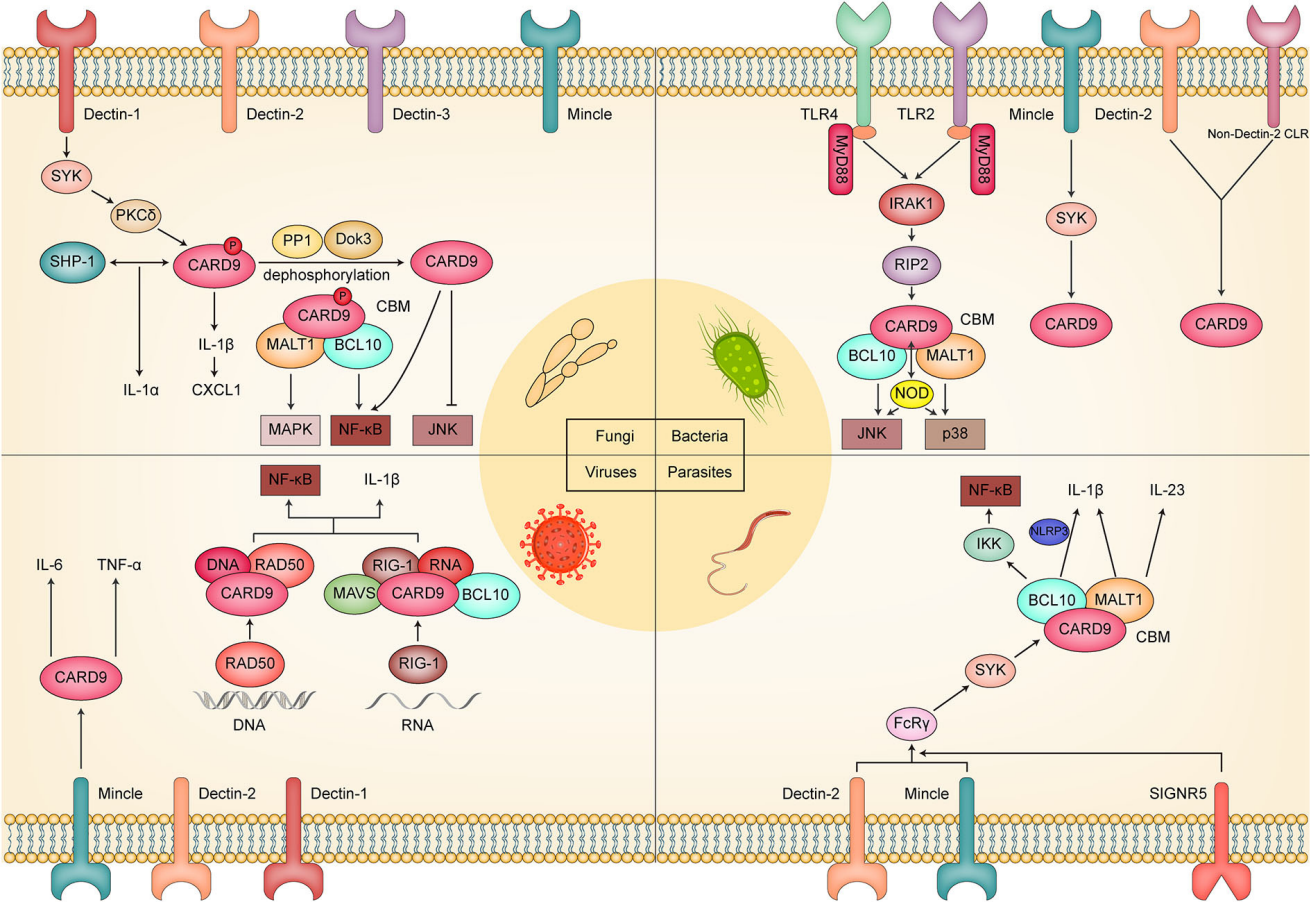
Schematic diagram of the molecular mechanism by which CARD9 plays a critical role in fungal, bacterial, viral, and parasitic infection.

Considering the central role of CARD9 in the immune reaction against fungi, bacteria, viruses, and parasites (Figure 2), CARD9 has been proposed as a novel therapeutic and vaccine-developing target. Recently, Hung et al. (2018) designed a multivalent *Coccidioides posadasii* vaccine called “GCP-rCpa1,” which consists of recombinant Coccidioides polypeptide antigen (rCpa1), yeast cell wall particles, and oligonucleotide to strengthen the protective cellular immune response to fungal invasion. In their subsequent study, further investigation was conducted to reveal the fungal vaccine's protective effect and the underlying mechanism. They found that vaccinated mice showed the upregulation of proinflammatory cytokines (TNF-α, IL-6, IL-1β), which was connected with the activation of Dectin-1/CARD9 and Dectin-2/CARD9 signaling. Moreover, they reported that the macrophage production of inflammatory cytokines and the acquisition of Th cells, especially Th 17 cells, were impaired in vaccinated Dectin-1^−/−^, Dectin-2^−/−^, and CARD9^−/−^ mice. Therefore, the mechanism by which GCP-rCpa1 activates the CLRs/CARD9 signaling pathway to initiate a potent Th 17 immune response was confirmed (Campuzano et al., 2020). Based on these, a more microbe-specific vaccine may be designed and developed by referring to the pattern of the GCP-rCpa1 vaccine. Regarding treatment, Kottom et al. (2020a) proposed that inhibiting the activity of CARD9 by pharmacological inhibitor BRD5529 might act as an underlying treatment strategy for *Pneumocystis jirovecii via* macrophage innate immune and anti-inflammatory activity. We have evidence that BRD5529 dramatically inhibited phospho-p38 and phospho-pERK1 signaling and the release of TNF-α upon *Pneumocystis* β-glucans exposure. In addition to BRD5529, currently known CARD9 inhibitors include BRD4203, BRD8991, and BRD4098 (Leshchiner et al., 2017), and they may also have the potential to become CARD9-targeted therapeutic drugs.

## Perspective and prospective direction

Infections driven by microbial pathogens cause more than 400 million deaths worldwide each year, a higher burden than either cancer or cardiovascular disease (GBD 2016 Causes of Death Collaborators, 2017). Innate and adaptive immunity are necessary for host homeostasis and are two critical ways to remove invasive microbial pathogens. Therefore, the essential role of the immune response induced by PRRs signaling has gained much attention, and a crucial downstream adaptor molecule called “CARD9” is widely involved in this cascade. At present, CARD9 has shown an emerging role in the infection immunity of fungi, bacteria, viruses, and parasites; it is partly based on the results of CARD9 KO mice experiments (Table 2). However, there is still some controversy surrounding its mechanism and effect in this regard. For example, Syk-CARD9 signaling is known as the classic CARD9-associated way to resist fungal invasion. However, Syk-dependent and CARD9-independent, plus Syk-independent, and CARD9-dependent specific antifungal signals, are proven to play an important role in candidiasis infection as well (Zajta et al., 2021). Therefore, future studies must explore the inner relationship between specific and classic pathways more definitively. In addition, is CARD9-induced susceptibility to microbial infection widely applicable to these four species? Indeed, CARD9 deficiency is currently reported to induce susceptibility to bacteria in an animal model but not in a human organism. Standing at a crossroads between immunity and infection, CARD9 is worth studying to elucidate the commonalities and individualities of immune defense in the infectious process of fungi, bacteria, viruses, and parasites. Therefore, CARD9 may offer an ideal opportunity to improve our understanding of the process of microbial infection in the future.

**Table 2:** Effects of CARD9-deficient mice on microbial infection.

**Microbe**	**Pathogens**	**Receptors**	**Effects**	**Organ involvement**	**References**
Fungi	*Pneumocystis*	Dectin-1/Mincle	Impaired proinflammatory response/cell differentiation	Lung	Kottom et al., 2020b
	*Mucor irregularis*	CLRs	Impaired cytokine and chemokine production	CNS	Sun et al., 2021
	*Rhizopus arrhizus*	PRRs	Impaired local cytokine production/Th cells response	Inguinal lymph nodes	Sun et al., 2019
	*Candida albicans*	CLRs	Impaired cytokine and chemokine production	CNS	Drummond et al., 2019
Bacteria	*Streptococcus pneumoniae*	Dectin-2	Decreased neutrophil accumulation	Lung	Ishizuka et al., 2020
Viruses	LACV	Dectin-1/Dectin-2/Mincle	Impaired cytokine production	Dendritic cells	Monteiro et al., 2019
	TMEV	CLRs	Increased loss of neuronal protein	Brain	Pavasutthipaisit et al., 2021
	CVB3	CLRs	Impaired cytokine production	Myocardium	Sun et al., 2020
Parasites	*Plasmodium berghei*	TLRs	Elevated proinflammatory response	Brain	Hafalla et al., 2012

LACV, La Crosse Virus; TMEV, Theiler's murine encephalomyelitis virus; CVB3, Coxsackievirus B3; CLRs, C-type lectin receptors; PRRs, pattern recognition receptors; TLRs, toll-like receptors; CNS, central nervous system.

## Author contributions

AH, ZH, and HZ wrote the first draft of this article. JZha, DZ, and HW were responsible for searching the scientific literature and drawing the schematic diagram. JZho and BC strictly reviewed and edited the manuscript. All authors approved the final version submitted and agreed on its submission to this journal.

## Conflict of interest

The authors declare that the research was conducted in the absence of any commercial or financial relationships that could be construed as a potential conflict of interest.

## Publisher's note

All claims expressed in this article are solely those of the authors and do not necessarily represent those of their affiliated organizations, or those of the publisher, the editors and the reviewers. Any product that may be evaluated in this article, or claim that may be made by its manufacturer, is not guaranteed or endorsed by the publisher.
